# A commentary on ‘Timing of restoration of bowel continuity after decompressing stoma, in left-sided obstructive colon cancer: a nationwide retrospective cohort’

**DOI:** 10.1097/JS9.0000000000001520

**Published:** 2024-05-03

**Authors:** Shuai Liu

**Affiliations:** Department of Gastrointestinal Surgery, Taizhou Hospital of Zhejiang Province, Affiliated to Wenzhou Medical University, Taizhou, People’s Republic of China

*Dear Editor,*

Recently, we read an article by Zamaray *et al*.^1^ on the timing of bowel continuity recovery after decompression stoma in left-sided obstructive colon cancer (LSOCC). Commendably, this is a nationwide retrospective cohort study with strong representativeness. The large cohort of diverse patient populations from 75 hospitals has enhanced the universality of the study results. This study also builds upon a strong preliminary research foundation, which conducted an in-depth exploration of LSOCC patients^2^. The comprehensive analysis of timing outcomes greatly enriches our understanding of the specificity of LSOCC. However, there are still certain details warranting further exploration.

Firstly, most patients with left-sided colon cancer presenting with acute obstructive symptoms are in the advanced or locally advanced stage. The evaluation of tumor (T) staging is often between T3 and T4. The author fails to elucidate the follow-up treatment for these patients’ post-decompressing stoma. Typically, such patients usually undergo conversion therapies, including neoadjuvant chemotherapy, radiochemotherapy, and, in some cases, targeted therapy and immunotherapy. The impact of these varied conversion therapies on colonic anastomosis healing differs, maybe even related to the risk of anastomotic leakage. After undergoing decompressing stoma to resolve acute obstruction, LSOCC patients need to be evaluated for clinical staging before determining the suitability of conversion therapies in the future. Therefore, the timing study for restoration of bowel continuity (ROBC) holds significant clinical guidance value, especially for LSOCC patients who have undergone conversion therapy.

Secondly, the surgical approach for left-colon cancer varies depending on the tumor location. Meanwhile, blood supply is an important factor affecting the anastomosis healing of laparoscopic colon cancer surgery^3^. For instance, whether the left colonic artery (LCA) needs to be preserved during surgery in distal sigmoid colon cancer and whether the superior rectal artery (SRA) needs to be preserved during surgery in proximal sigmoid colon cancer. In theory, patients with preserved LCA and SRA during surgery have a more sufficient residual intestinal blood supply, and the risk of anastomotic leakage is lower. Effective preservation of LCA and SRA may reduce the incidence of anastomotic leakage. The above two situations may influence the short-term prognosis and complications after ROBC, requiring a subgroup analysis.

Thirdly, nutritional risk assessment among LSOCC patients should be included in the study. Malnutrition has been recognized as a risk factor for postoperative complications. Nutritional support therapy for surgical patients includes correcting preoperative undernutrition and maintaining postoperative nutritional status. The meta-analysis indicates that immunonutrition may reduce the incidence of total postoperative complications, including anastomotic leakage, in adult patients undergoing elective surgery for gastrointestinal cancer^4^. In addition, the surgical procedure stress response could promote skeletal muscle catabolism, exacerbating negative nitrogen balance^5^. Therefore, individual perioperative nutritional status needs to be assessed, and tailored nutritional interventions for different nutritional risks need to be mentioned.

We sincerely appreciate the authors’ timing study on bowel continuity after decompressing stoma in LSOCC. However, further consideration of the details of different conversion treatments, blood supply, and perioperative nutrition could further refine the study and enhance clinical guidance.

## Ethical approval

Not applicable.

## Consent

Not applicable.

## Source of funding

Science and Technology Plan Project of Taizhou (22ywb07).

## Author contribution

T.H.: investigation and writing – original draft; S.L.: conceptualization, funding acquisition supervision, and writing – review and editing.

## Conflicts of interest disclosure

The authors declares no conflicts of interest.

## Research registration unique identifying number (UIN)

Not applicable.

## Guarantor

Shuai Liu.

## Data availability statement

Not applicable.

## Provenance and peer review

Not applicable.
